# Species-Specific Seed Dispersal in an Obligate Ant-Plant Mutualism

**DOI:** 10.1371/journal.pone.0004335

**Published:** 2009-02-04

**Authors:** Elsa Youngsteadt, Jeniffer Alvarez Baca, Jason Osborne, Coby Schal

**Affiliations:** 1 Department of Entomology and W. M. Keck Center for Behavioral Biology, North Carolina State University, Raleigh, North Carolina, United States of America; 2 Facultad de Ciencias Biológicas, Universidad Nacional de San Antonio Abad del Cusco, Cusco, Perú; 3 Department of Statistics, North Carolina State University, Raleigh, North Carolina, United States of America; Queen Mary College, University of London, United Kingdom

## Abstract

Throughout lowland Amazonia, arboreal ants collect seeds of specific plants and cultivate them in nutrient-rich nests, forming diverse yet obligate and species-specific symbioses called Neotropical ant-gardens (AGs). The ants depend on their symbiotic plants for nest stability, and the plants depend on AGs for substrate and nutrients. Although the AGs are limited to specific participants, it is unknown at what stage specificity arises, and seed fate pathways in AG epiphytes are undocumented. Here we examine the specificity of the ant-seed interaction by comparing the ant community observed at general food baits to ants attracted to and removing seeds of the AG plant *Peperomia macrostachya*. We also compare seed removal rates under treatments that excluded vertebrates, arthropods, or both. In the bait study, only three of 70 ant species collected *P. macrostachya* seeds, and 84% of observed seed removal by ants was attributed to the AG ant *Camponotus femoratus*. In the exclusion experiment, arthropod exclusion significantly reduced seed removal rates, but vertebrate exclusion did not. We provide the most extensive empirical evidence of species specificity in the AG mutualism and begin to quantify factors that affect seed fate in order to understand conditions that favor its departure from the typical diffuse model of plant-animal mutualism.

## Introduction

**Figure 1:**
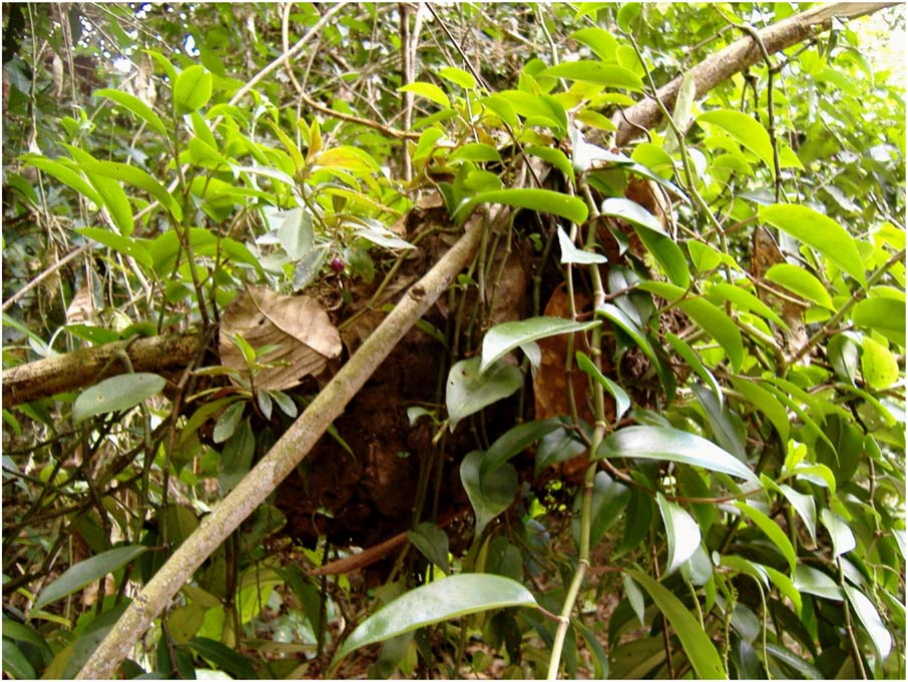
Ant garden in southeast Perú. This nest houses the ants *Camponotus femoratus* and *Crematogaster levior* and the epiphytic plants *Peperomia macrostachya* and *Codonanthe uleana* Fritsch (purple fruit). Such gardens are established when ants embed seeds of AG epiphytes into their arboreal carton nests.

To survive, seeds must arrive at suitable germination sites. This poses special problems for epiphyte seeds, which must move against gravity to arrive at very specific and patchy germination sites. The vast majority of epiphytes meet these requirements by producing abundant wind-dispersed diaspores or by attracting vertebrate frugivores likely to deposit seeds in feces on branches above the ground [1], [2]. A small but conspicuous minority of epiphytes rely upon ants for dispersal. Throughout the Amazon basin, this strategy is represented by some 15 epiphyte species that grow exclusively or principally in arboreal carton nests built by ants, forming abundant hanging gardens known as ant-gardens (AGs) (Fig. 1) [3]–[6]. In this habitat, epiphytes are limited by substrate and nutrient availability, and AGs are considered the most important substrate for vascular epiphytes due to their porous texture and enriched N, K, and P relative to other insect carton or surrounding soil [7], [8]. AG epiphytes further rely upon ants for defense against herbivores and for seed dispersal [5], [9], [10].

Ant-gardens are notable not only as the product of an unusual seed dispersal strategy in epiphytes, but also as the most complex form of ant-plant symbiosis [11]. AGs are initiated when ants collect seeds of specific epiphytes and carry them to their nests, incorporating them into the carton walls [3], [5], [12]. AG ants collect the seeds in response to chemical cues and independently of nutritional rewards, removing them directly from the plants, from vertebrate feces and from the soil surface [3], [5], [13], [14]. The ants rely upon the roots and leaves of the germinated plants for nest structure and dehumidification; without epiphytes, the carton nests disintegrate during the rainy season [15]. The AG mutualism also makes it possible for the ants to colonize resource rich microhabitats independently of pre-existing nest substrates, an advantage that may have led to the dominance of AG ants in lowland Amazonia [5], [16]. In southeastern Perú, AG territories occupied 16% to 39% of a 12 km transect, depending on habitat type [5]. Further, in those same forests AG ants are the most frequently encountered, numerically abundant and behaviorally dominant species in arboreal ant samples and at terrestrial baits [5], [16].

The AG flora and fauna are taxonomically diverse, but specific and consistent through time and space. AG-restricted epiphytes occur in seven different plant families, and AG construction has been confirmed in four ant species in three subfamilies, all of which represent independent origins of traits necessary for the AG symbiosis [5], [6], [17]. Although the AG interaction involves more than two partners, its specificity is nonetheless in contrast to the frequently diffuse nature of plant-animal interactions, such as seed dispersal and many pollination mutualisms, that inform current understanding of mutualism [18]–[21], [but see 22]. It is therefore of interest to elucidate the mechanisms that favor and maintain the pattern of specificity in this seed-dispersal mutualism.

Some AG seeds bear adhering fruit pulp, oils, or lipid-rich elaiosomes, which could motivate seed collection by multiple ant species, but published observations support some level of specialization in the ant-seed interaction. Orivel and Dejean [3] demonstrated that the AG ants *Camponotus femoratus* (Fabricius) and *Pachycondyla goeldii* (Forel) collect seeds of AG epiphytes even when fruit pulp and elaiosomes have been removed. *C. femoratus* did not carry seeds of non-AG congeners of AG plants [5, EY pers. obs.]. On the other hand, Davidson [5] presented seeds of three species of AG plants, with putative food rewards intact, to single colonies of four generalist non-AG ant species; three of those ant species (*Camponotus sericeiventris* (Guérin-Méneville), *Dolichoderus attelaboides* (Fabricius) and *Cephalotes spinosus* (Mayr)) did not carry AG seeds, while a fourth (*Dolichoderus bidens* (Linnaeus)) did. Thus 25% of non-AG ants were observed to carry the AG seeds, but it is unclear, based upon this small sample size of ant species and colonies, what degree of specificity would be expected in the ant community at large. In addition to the role of ants in AG seed dispersal, many other possible influences on AG seed fate are unknown.

We can conceive of three explanations for the AG-restricted distribution of AG epiphytes: (1) only AG ants are attracted to and collect AG seeds; (2) other ants are attracted to AG seeds but are excluded from collecting them by the abundant and dominant AG ants; and (3) other organisms such as mammals or non-AG ants also collect the seeds but destroy them or deposit them in locations unsuitable for plant survival. Here, we distinguish among these alternatives by comparing the community of ants that could potentially interact with seeds (i.e., ground-foraging species detected at general food baits) to those actually visiting and removing seeds of the abundant AG plant *Peperomia macrostachya* (Vahl). We further address factors that influence dispersal and predation of *P. macrostachya* seeds by comparing seed removal rates under selective exclusion of vertebrates, arthropods, neither, or both. Finally, we present an estimate of seed survival for those seeds that are finally retrieved to AG carton.

## Results

### Bait study

Of ant species that could have potentially interacted with AG seeds, very few actually did so. Ants were observed at 105 (97%) of the 108 terrestrial bait stations when baited with food and only at 28 (26%) of the stations when baited with *P. macrostachya* seeds. At 20 of these 28 stations (71%), seeds were visited only by the AG ant *C. femoratus*, or *C. femoratus* together with its heterospecific nestmate *Crematogaster levior* Longino. Although *C. femoratus* was also the single most common visitor to food baits, it accounted for a much lower proportion of visited food baits than seed baits: 26 of 105 stations (25%). Seventy ant species were collected overall: 68 at food baits and eight at AG seed baits. Most baits hosted one species at a time, with a maximum of five species collected during a single observation. Only three of the eight species at AG seeds were observed to collect the seeds (Table 1), and multiple workers of all three species removed seeds in an apparently “purposeful” manner, grasping seeds from the tray and walking quickly away with them, continuing to carry them until disappearing into leaf litter or dense vegetation where we did not follow. The conditional probability of *C. femoratus* appearing at seeds given its presence at a bait station differed significantly from the same conditional probability of non-AG ants (χ^2^ = 31.3, df = 1, *P*<0.0001). The odds ratio for these conditional probabilities was (21/9)/(18/86) = 11.1, with a 95% CI of 4.4 to 28.3. In other words, when present at a bait station, *C. femoratus* was 11.1 times more likely than other ants to appear at the seed bait.

**Table 1:** Ant species recorded at general food baits and at ant-garden seed baits on transects in southeast Perú.

Classification		Number of food baits visited	Number of seed baits visited	Classification		Number of food baits visited	Number of seed baits visited
**Ponerinae**				36	*Pheidole* sp. 5	1	
1	*Ectatomma tuberculatum*	3		37	*Pheidole* sp. 6	1	
2	*Ectatomma lugens*	5		38	*Pheidole* sp. 7	1	
3	*Gnamptogenys* sp. (*striatula* group)	1		39	*Pheidole* sp. 8	1	
4	*Gnamptogenys moelleri*	1		40	*Pheidole* sp. 9	1	
5	*Odontomachus laticeps*	1		41	*Pheidole* sp. 10	1	
6	*Pachycondyla constricta*	1		42	***Sericomyrmex*** ** sp. 1**	**3**	**3**
7	*Pachycondyla crassinoda*	3		43	*Sericomyrmex* sp. 2	0	1
8	*Pachycondyla harpax*	1		44	*Solenopsis virulens*	5	
9	*Paraponera clavata*	2		45	*Solenopsis-2*	7	
**Myrmicinae**				46	*Solenopsis-3*	1	
10	*Apterostigma auriculatum*	1		47	*Trachymyrmex farinosus*	1	
11	*Apterostigma* sp. (*pilosum* group)	1		48	*Trachymyrmex* cf. *bugnioni*	1	
12	*Crematogaster brasiliensis*	9	1	49	*Trachymyrmex* sp.	1	
13	*Crematogaster levior*	23	8	50	*Wasmannia auropunctata*	6	
14	*Crematogaster limata*	3		**Formicinae**			
15	*Crematogaster sotobosque*	3		51	*Brachymyrmex* cf. *longicornis*	4	
16	*Crematogaster tenuicula*	7		52	*Camponotus indianus*	1	
17	*Megalomyrmex balzani*	3		53	*Camponotus amoris*	1	
18	*Ochetomyrmex neopolitus*	2		54	*Camponotus atriceps*	1	
19	*Ochetomyrmex semipolitus*	3		55	*Camponotus cacicus*	3	
20	*Pheidole astur*	9		56	***Camponotus femoratus***	**26**	**21**
21	*Pheidole biconstricta*	2		57	*Camponotus lespesi*	4	
22	*Pheidole deima*	1		58	*Camponotus novogranadensis*	2	
23	*Pheidole embolopyx*	1		59	*Camponotus sericeiventris*	4	1
24	*Pheidole fimbriata*	1		60	*Camponotus* sp. cf. *cressoni*	3	
25	*Pheidole laidlowi*	3		61	*Camponotus* sp. 1	1	
26	*Pheidole nitella*	2		62	***Camponotus*** ** sp. 2**	**1**	**2**
27	*Pheidole scolioceps*	1		63	*Camponotus* sp. 3	1	
28	*Pheidole xanthogaster*	1		64	*Paratrechina* sp. 1	1	
29	*Pheidole* sp. (nr. *deima*)	1		65	*Paratrechina* sp. 2	2	
30	*Pheidole* sp. (nr. *leptina*)	1		**Dolichoderinae**			
31	*Pheidole* sp. (nr. *peruviana*)	2		66	*Dolichoderus attelaboides*	1	
32	*Pheidole* sp. 1	1		67	*Dolichoderus imitator*	0	1
33	*Pheidole* sp. 2	1		68	*Dolichoderus bispinosus*	1	2
34	*Pheidole* sp. 3	1		**Pseudomyrmicinae**			
35	*Pheidole* sp. 4	1		69	*Pseudomyrmex tenuis*	1	
				70	*Pseudomyrmex* sp. cf. *tenuis*	4	

Food baits consisted of canned tunafish and strawberry jam; seed baits were *P. macrostachya* seeds.

Ant species that collected *P. macrostachya* seeds are in bold type.

**Figure 2:**
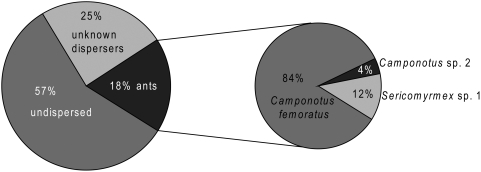
Fate of *P. macrostachya* seeds placed at 108 sampling stations. Most *P. macrostachya* seeds remained undispersed during the bait study. Three ant species removed 390 seeds, and the AG ant *C. femoratus* was responsible for the vast majority of observed dispersal.

Of 2205 seeds presented at bait stations, 43% (938 seeds) disappeared during the course of the observations. Ants accounted for 42% of seeds removed (390 seeds). *C. femoratus* was responsible of the overwhelming majority of ant-removal of AG seeds (Fig. 2). *Sericomyrmex* sp. 1 (determined to genus by T. Schultz) and *Camponotus* sp. 2. (determined by W. Mackay as an undescribed species) were also observed to carry seeds (Fig. 2). (Though unobserved ants may have removed some seeds, we restrict these results and subsequent discussion only to observed interactions.) Cockroaches and crickets were often found at baits, and seeds sometimes clung to their legs or were dislodged from the tray.

Ants that occurred at seed baits without collecting seeds engaged in various behaviors, none of which appeared to be direct use of seeds. *C. levior* occurred at seed baits only with *C. femoratus*, with which it shares nests and foraging trails. Although *C. levior* was observed foraging alone (independently of *C. femoratus*) at food baits, it never appeared to forage independently for seeds, and made no visible attempt to remove seeds. *Crematogaster brasiliensis* Mayr, a common species at food baits, appeared at only one seed bait and did not interact directly with seeds but appeared to investigate the plastic tray itself rather than the seeds. *Camponotus sericeiventris* was represented at seed baits by a single individual which repeatedly antennated both plate and seeds and displayed alarm-like behavior. *Dolichoderus imitator* Emery and *Sericomyrmex* sp. 2 were also represented by single individuals, apparently exploring. *Dolichoderus bispinosus* (Olivier) appeared at two seed baits where it investigated seeds; four seeds disappeared from one bait where this species was found during the morning observation, but no further seeds were removed during 20 min of direct observation or when the bait was checked again 30 min after that. By the afternoon observation, *D. bispinosus* had been replaced at the bait by *C. femoratus*.

### Exclusion experiment

**Figure 3:**
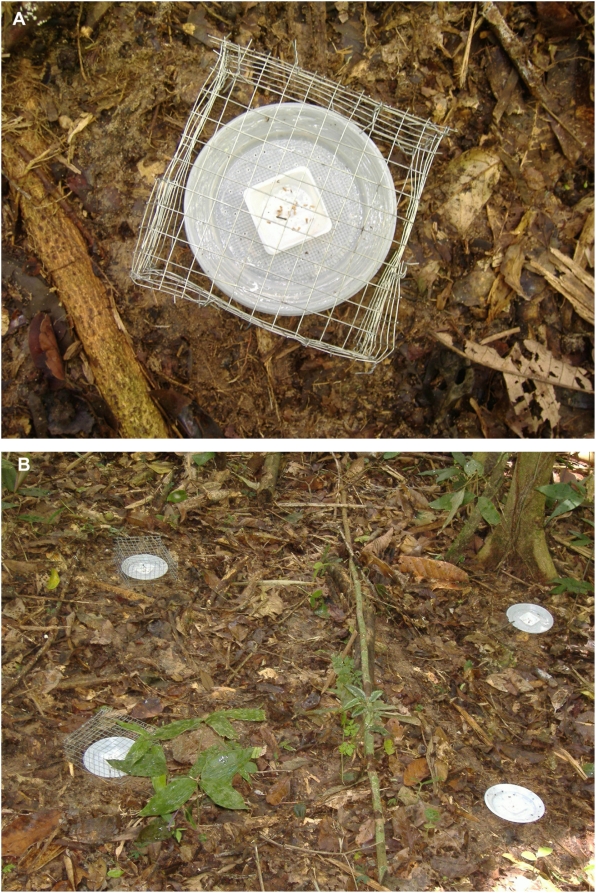
Arthropod and vertebrate exclusion experiments. (A) Close-up of a seed tray treated with both Tanglefoot and vertebrate-exclusion cage. (B) Experimental design showing the four treatments presented in random positions in 1 m^2^ plots.

**Figure 4:**
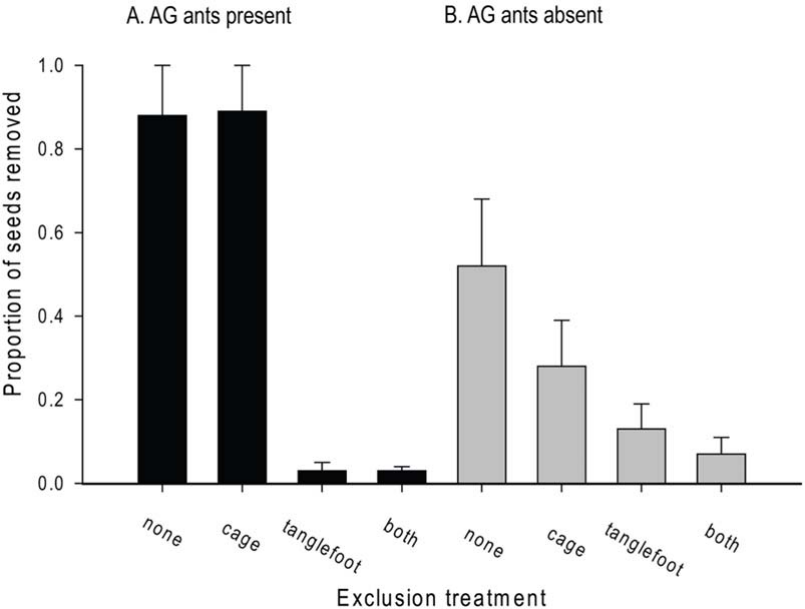
Removal of *P. macrostachya* seeds during exclusion experiments. Bars are proportion of seeds removed from each treatment±SE, based on untransformed counts of seeds removed. Black bars (A) represent plots placed within AG territories, and gray bars (B) represent plots placed outside of AG territories. Each bar represents results for 240 seeds (15 seeds per exclusion treatment per block per day, over four blocks and four days).

Arthropod exclusion (using Tanglefoot: Fig. 3) significantly decreased the number of *P. macrostachya* seeds removed from the seed plates (Fig. 4, Table 2). The effect of *C. femoratus* on seed removal was significant only in the absence of Tanglefoot; where AG ants were present, many more seeds were removed from Tanglefoot-free plates than Tanglefoot-treated plates (Fig. 4). In AG territories, many *C. femoratus* became trapped in Tanglefoot, which had to be cleaned often to prevent foragers reaching seeds by walking on trapped ants. In non-AG territories, Tanglefoot occasionally trapped an apparently idiosyncratic variety of insects but not ants.

**Table 2:** Results of ANOVA testing for fixed effects of cage, Tanglefoot and *C. femoratus* (“AG ants”) in the exclusion experiment.

Source	Numerator DF	Denominator DF	Sum of squares	*F*-ratio	*p*-value
*Main effects*					
Cage	1	18	0.99	0.46	0.506
Tanglefoot	1	18	168.87	78.56	**<0.001**
AG ants	1	3	24.60	5.28	0.105
*Interactions*					
Cage×Tanglefoot	1	18	0.99	0.46	0.505
Cage×AG ants	1	18	3.23	1.50	0.105
Tanglefoot×AG ants	1	18	49.94	23.23	**<0.001**
*Simple effects of AG ants*					
AG ants, Tanglefoot absent	1	5.15	72.32	18.21	**0.006**
AG ants, Tanglefoot present	1	5.15	2.22	0.56	0.485
Cage×Tanglefoot×AG ants	1	18	0.08	0.04	0.846
*Random effects*					
Blocks	3	.	10.58		
Blocks×AG ants	3	.	17.38		
Residual	18	.	38.69		
*Corrected total*	31	.	315.36		

Vertebrate exclusion (wire mesh cages) had no significant effect on seed removal, although cages tended to diminish seed removal in non-AG territories. A total of 34 undispersed seeds appeared to have been chewed or crushed and left on the plates. Twenty nine of these were left at two non-AG plots, and five in a single AG plot. The unknown culprit(s) accessed seeds in all exclusion treatments without disturbing cage placement, and is likely to be an arthropod capable of jumping and/or flying.

### Seed fate in AG carton

We observed a total of 794 *P. macrostachya* plants in 10 AGs. Of these, 91% (720 plants) were recently germinated seedlings, 2% (18) were juvenile and 7% (56) were mature plants. Given our three assumptions (see Methods), this census yields a maximum seedling-to-adult transition probability of 8%.

## Discussion

We present the strongest available evidence that the AG ant-seed interaction is highly specific in lowland forest of the Peruvian Amazon, and thus represents an exception to the general understanding of seed-dispersal mutualisms as generalized and diffuse interactions [18], [21], [23], [24]. Ants that removed *P. macrostachya* seeds were a very small subset of the generalist ground-foraging fauna, and the AG ant *C. femoratus* was by far the most abundant and persistent remover, and probable disperser, of AG seeds. Common granivorous species were conspicuously absent. We therefore reject the hypothesis that competition between ant species or post-dispersal events limit *P. macrostachya* seeds to AGs. Instead, the distribution of *P. macrostachya* in AG ant nests arises largely due to specificity of the ant-seed interaction, and probably also due to seed or seedling death when removed by other species.

### Factors affecting *P. macrostachya* seed fate

**Figure 5:**
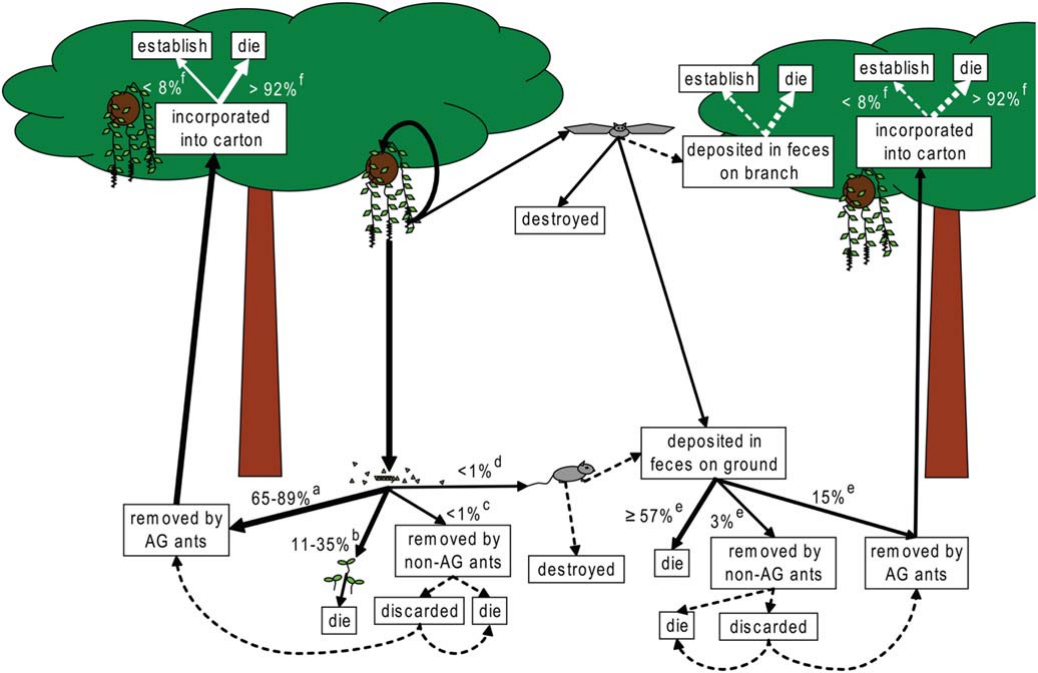
Actual and putative seed fate pathways for *P. macrostachya* seeds. Estimated probability of events documented in the bait study, exclusion experiment, and garden census are noted by percent values next to solid lines (see footnotes below). Solid lines lacking percent values have been reported anecdotally in the literature or observed by E.Y., while pathways represented by dotted lines are proposed but undocumented. Seeds may be dispersed directly from AG plants by both ants and mammals, or they may fall to the ground. Seeds on the ground in AG territories are retrieved primarily by *C. femoratus*. If seeds undergo long distance dispersal, as when they are consumed by flying or arboreal mammals, they may also be deposited on the forest floor where they can die, be retrieved by non-AG ants or AG ants far from the original colony. Incorporation into AG carton represents a seed's best, but still unlikely, opportunity for survival. Sources of percent values: a. 65% of *P. macrostachya* seeds were removed from within AG territories in the one-day bait study; 89% of *P. macrostachya* seeds were removed from within AG territories in the four-day exclusion experiment. b. 35% of seeds were not removed from within AG territories in the bait study; 11% were not removed in the exclusion experiment. We assume that un-removed seeds germinate in place and die. c. In the bait study, we did not observe non-AG ants removing *P. macrostachya* seeds within AG territories. However, *Dolichoderus bispinosus* may have done so, and we have occasionally seen *Pheidole astur* removing *P. macrostachya* seeds from near *C. femoratus* foraging trails. d. In the exclusion experiment, 3% of seeds were removed from trays treated with Tanglefoot, and from trays treated with both Tanglefoot and mesh cage, suggesting that mammals were of minimal importance in seed removal from AG territories. e. Here we assume that seeds deposited in vertebrate feces would occur randomly inside and outside AG territories, and would be treated the same way as seeds that had not passed through a digestive system. Hence we apply the numbers from our bait study, including bait stations inside and outside of AG territories. f. Numbers taken from our estimate of seed survival upon arrival in AG carton.

The actual and putative seed fate pathways we propose for *P. macrostachya* are represented graphically in Fig. 5. These pathways include seed removal by *C. femoratus*, other ants, and vertebrates. The few removal events attributable to ants other than *C. femoratus* (less than 3% of all seeds presented at baits) were unlikely to result in successful germination. The natural history of *Camponotus* sp. 2 (Table 1) is unknown; it occurred once at a food bait and twice at seed baits, each time at night in or near bamboo thickets. *Sericomyrmex* and other lower attine species have been previously reported as important secondary dispersers of seeds in various tropical habitats, where they retrieve typically vertebrate-dispersed seeds with adhering fruit. The cleaned seeds are either retained in fungus gardens or later discarded in viable condition in rubbish heaps [25], [26]. It is unclear whether the *Sericomyrmex* observed in this study would keep *P. macrostachya* seeds in the nest for fungus-culturing, or discard them. In either case, because *Sericomyrmex* nests on the ground and AG species succeed only in the canopy, the seeds would be doomed or would await further dispersal. All instances of non-AG ants removing *P. macrostachya* seeds took place at bait stations where *C. femoratus* did not occur. Nevertheless, casual observations of *Pheidole astur* Wilson retrieving seeds near *C. femoratus* foraging trails indicates that it is not impossible for other species to collect the seeds even within AG territories (Fig. 5). The non-AG ant *Dolichoderus bidens* has also been observed to carry AG seeds, including *P. macrostachya*, when directly confronted with the seeds [5], but *D. bidens* was not observed in the present study.

Both the bait study and the exclusion experiment provide indirect support for our assertion that *C. femoratus* is the main disperser of AG seeds in the study area. The major effect of Tanglefoot both within and away from AG territories further suggests that the unobserved seed removers in both experiments are likely to be arthropods rather than vertebrates. The absence of a vertebrate effect is unusual but not unprecedented with small tropical seeds [27]–[29]. A vertebrate effect could have been masked by the experiment itself if small vertebrates were deterred by the presence of plastic plates and/or Tanglefoot. Our experiments do not address the possibility of vertebrates removing seeds directly from the AG plants. This phenomenon has rarely been observed, but Davidson [5] noted *P. macrostachya* seeds in bat droppings and reported both birds and monkeys feeding on fruits of other AG epiphytes. Sticky *P. macrostachya* seeds may also be dispersed by adhering to vertebrates that visit AGs to consume other fruits [13]. *C. femoratus* likely removes AG seeds from vertebrate feces, so vertebrate consumption does not categorically doom a seed, and may represent one of few opportunities for long distance dispersal in a system where ants usually return seeds to the garden of origin or to a neighboring garden of the same ant colony [5]. Occasional dispersal to suitable sites by arboreal vertebrates may also have led to the establishment of the few *P. macrostachya* plants observed to grow independently of AGs. Finally, there is some evidence that ants may later build carton nests around AG plants that establish independently [30, G. Mathieu personal communication]. This phenomenon has yet to be thoroughly documented, and has not been observed in Perú (E.Y. personal observation, D. Davidson, personal communication) but deserves further attention, and could be critical to *P. macrostachya* fate and distribution in some regions. There may be a role for all these pathways in determining AG seed fate, but their importance requires further investigation (Fig. 5).

In the exclusion experiment, 88% of arthropod-accessible *P. macrostachya* seeds presented in AG territories were removed from the plates. In the bait study, we observed a lower overall level of seed removal in AG territories: 65% of seeds were removed from bait stations where *C. femoratus* was observed at least once at either food or seeds. The discrepancy is probably due to at least two differences in seed presentation between the two studies. In the exclusion experiment, seeds were intentionally located centrally in AG foraging territories, and were present for four consecutive days. In the bait study, stations were located without respect to AG territories and seeds were present for only one day, so that some bait stations were peripheral to AG territories, visited by few ants, and/or discovered only toward the end of the observation period. In both experiments, seed removal was much lower in the absence of AG ants: 52% and 31%, respectively.

When seeds do arrive in AG carton, the probability of survival is still low. Ants incorporate hundreds of seeds into the carton of even a single nest, which can only support a few adult plants. Even in a single snapshot census, these seedlings are almost 13 times more abundant than mature plants on gardens; it is likely that some retrieved seeds do fail to germinate, and that there is some seedling turnover on the nest during the *P. macrostachya* fruiting season. Therefore, our estimate of seedling survival is probably an upper bound, and mortality must approach 100% as seedlings are winnowed and few adult plants establish. Nevertheless, the estimated survival rate of ≤8% is comparable to seed or seedling survival rates measured in other ant-dispersed seeds and other epiphytes: survival rates of either seeds that have already arrived at ant nests or potentially suitable branches, or of young seedlings that have just germinated in such locations, can range from about 3% to 30% [e.g. 31]–[35]. For AG plants, establishment opportunities may occur mainly when carton is added to existing nests or when new nests are initiated.

Nevertheless, many seed fate pathways remain unexplored in the AG system. Though they did not appear in the present study, other ant species are known to carry AG seeds and build gardens. *Azteca* spp. sometimes carry *P. macrostachya* seeds [5], while the gardening species *Pachycondyla goeldii* and *Odontomachus mayi* Mann rarely carry or cultivate *P. macrostachya*, instead demonstrating strong preference for other AG plants [3]. The relative importance of these other gardening species to seed fate in *P. macrostachya* and other AG seed species remains to be determined. Future work should also investigate agents of long-distance dispersal and their contribution to gene flow in AG plants, compare patterns of seed movement among the taxonomically diverse AG epiphytes, and assess factors affecting seed fate over a wider geographic range.

### Why don't other ants collect *P. macrostachya* seeds?

Further evidence of species-specificity in the *P. macrostachya*-*C. femoratus* interaction comes not from direct observation of seed movement, but from the conspicuous absence of ants that might ordinarily collect seeds. For instance, the extremely diverse genus *Pheidole* includes many granivorous species and, to our knowledge, is reported at seeds in every systematic study of small-seed dispersal and granivory in the New World tropics [26]–[28], [36]–[40]. Among the *Pheidole* species collected at food baits in the present study, at least *Pheidole fimbriata* Roger, *Pheidole nitella* Wilson and *Pheidole peruviana* Wilson are known or suspected granivores [40]. *P. astur* occasionally collected *P. macrostachya* seeds when these were presented to *C. femoratus* in behavioral assays for a different experiment. Although *P. astur* did exploit food in the bait study, it was not observed at seed baits so its retrieval of AG seeds could not be quantified. The absence of *Pheidole* ants at *P. macrostachya* seed baits, despite its detection at 36 general food baits, suggests that these seeds may repel or deter at least some ant species.

In addition to the scarcity of granivores at seed baits, comparison with other tropical ant-seed interaction studies suggests that *P. macrostachya* seeds are exceptionally under-visited by ants. In a study of ants using six different nonmyrmecochorous seed species in Brazil, Pizo and Oliveira [28] found that 90% of the surveyed seeds were attended by ants at least once during six surveys in a 24 hour period even though these seeds did not offer specialized ant rewards. By comparison, in the present study only 26% of *P. macrostachya* seed baits were attended by ants during 3 surveys in the same time period, and only 17% by ants other than *C. femoratus*.

Seed size can inform which ant species utilize available seeds [26], [28]. It seems unlikely, however, that seed size is wholly responsible for the patterns observed in this study. At least half the ant species collected at food baits were clearly large enough that individual workers could have easily carried *P. macrostachya* seeds. Though other studies have found that *C. levior* attempts to carry AG seeds, but is unable to do so because of its small size or because it is displaced by *C. femoratus* [3], [5], we were unable to confirm or refute these observations in the present study. *C. levior* was often present at seed baits together with *C. femoratus*, but *C. levior* never foraged independently for seeds as it did for food, nor did it make visible attempts to carry seeds. Thus it is unclear whether *C. levior* arrived at *P. macrostachya* seed baits in this study because it is attracted to them, or whether its presence was an incidental result of shared trail use with *C. femoratus*.

Furthermore, if the seeds were attractive to other species, ants of any size should still have been observed interacting with the seeds even if not removing them. Instead, when we did find non-AG ants at AG seed baits, they appeared to ignore the seeds, or in the case of *C. sericeiventris*, to be alarmed by them—an outcome that has also been reported previously [5].

*P. macrostachya* seeds emit many phenolic and terpenoid volatiles, and the component geranyl linalool is shared among at least eight AG seed species [14, E.Y. unpublished data]. These components, though accepted by and even attractive to *C. femoratus*, could act as deterrents to other species; geranyl linalool in particular is toxic to many ants [41]. A related phenomenon occurs in flowering plants that produce ant-repellent floral scents [42], nectar [43], or pollen [44] that prevent detrimental activities of ants on flowers—namely, nectar theft and deterring pollinators. Floral ant-repellents may be particularly well developed in plants that are adapted to attract or house ant-guards [45], and there is evidence that, as we suggest for *P. macrostachya*, such floral repellents can be widely effective ant deterrents while still admitting one or a few ant species [46].

To confirm repellency of *P. macrostachya* seeds to non-AG ants, it would be interesting to conduct additional experiments comparing ants at AG seeds to ants utilizing alternative seed baits. We did not undertake comparison to other seeds in the present study because the a priori choice of alternative bait would have been problematic. The present results suggest that generalists, predators and granivores are all under-represented at *P. macrostachya* seeds, and these observations could be further tested by comparison to seeds that are known to attract such ants in other habitats—namely, lipid-rich, fruity or elaiosome-bearing seeds for generalists and predators [28], [47], and dry seeds such as barley for granivores [26], [48]. Seed extracts could also be tested for repellency to non-AG ants in an olfactometer assay [14], [41].

Other ant species reported as gardeners (*Odontomachus mayi*, *Pachycondyla goeldii*, and *Azteca* spp.) were not detected in the present study. A single *Pachycondyla* garden has been noted at the study site, and *Azteca* gardens, although they do host *P. macrostachya* plants, account for no more than 5% of gardens in terraza and bajío habitats at the site. *Azteca* species did not appear at food or seed baits in this study.

### Why specialize?

Overall, we describe an unusually specific and intimate seed-dispersal mutualism and provide the first empirical account of seed movements in an ant-garden epiphyte. Although the AG system is a case of interacting guilds rather than a one-to-one partnership—some 15 epiphyte species grow in gardens built by four ant species over the range of the interaction—the mutualism nonetheless contrasts with the current understanding of seed dispersal as a general interaction in which animal and plant partners interact in diffuse and asymmetrically dependent networks. Herrera [24] described factors that should limit specialization in seed-dispersal mutualisms, including unpredictability of germination sites in space and time, and weak reciprocal selective pressure by plants and dispersers. AGs, however, make suitable germination sites predictable. At the study site, *C. femoratus* appears to be the only ant capable of dispersing the seeds to suitable sites, and removal by other means will nearly always have negative consequences. It is also noteworthy that AG partners remain associated after the act of dispersal, throughout their life histories, and both plants and ants depend upon this intimate cohabitation for survival. They may therefore exert stronger and more consistent selective pressures upon one another than free-living mutualists.

This study, however, provides only a snapshot of the interaction in time and space. To clarify the selection pressures that promote or prevent coevolution in AG partners, future studies should compare the benefits (nutrients, protection, seed dispersal efficiency) conferred by different AG ant species that occur in other regions, and the seed traits to which those ants respond. For example, Youngsteadt et al. [14] identified a blend of volatile compounds from *P. macrostachya* seeds that attracted *C. femoratus*, and chromatographic fractions of *P. macrostachya* extract that elicited seed-carrying behavior. It is not known whether these same seed characteristics are responsible for the behavior of all AG ants, or whether different species may exert conflicting selective pressures upon the seeds. Similarly, AG ants interact with multiple plant partners. Whether the specificity and selective pressures in the *C. femoratus-P. macrostachya* mutualism are duplicated in all AG ant-seed interactions remains to be determined.

## Materials and Methods

### Field site and study species

Studies were conducted during October through December, 2006, at the Centro de Investigación y Capacitación Río Los Amigos in Madre de Dios, Perú (located at 12°34′07″S, 70°05′57″W) consisting of floodplain forest (bajío), upland forest (terraza) and bamboo thickets (pacal). AGs constructed by the ant *Camponotus femoratus* are abundant in both the bajío and terraza habitats, with aggregations of 2–30 nests occurring along trails in those habitats at an average interval of about 300 m (E.Y. unpublished data). *C. femoratus* occupied more than 95% of AGs in these habitats (*n* = 168 AGs censused), and 98% of *C. femoratus* nests also housed the parabiotic ant *Crematogaster levior*. The other AGs at the site were constructed by *Azteca* species. Nine species of epiphytes regularly occur in AGs at the field site; most (56%) of the 162 *C. femoratus* gardens surveyed hosted a single plant species and 44% hosted two or more plant species, occasionally up to six or seven (E.Y. unpublished data). The most abundant is *Peperomia macrostachya*, which occupies 91% of gardens at the site and which Davidson [5] described as an AG pioneer species, among the first to grow in newly established gardens. This species is considered AG-restricted, rarely occurring outside of ant nests; of 674 *P. macrostachya* plants observed at a nearby site [5], only 5 individuals grew independently of AGs. We therefore assumed that *P. macrostachya* was a representative AG plant central to the AG mutualism, and used freshly collected mature *P. macrostachya* seeds in all seed removal experiments described below. All seeds were collected with forceps and transported in clean Petri dishes.

### Bait study

We surveyed the ant assemblage at 108 sampling stations placed every 25 or 50 m along sections of the established trail system, 1 m off the trail and randomly assigned to the left or right of the trail. Of the 108 stations, 27 were in bajío habitat, 69 in terraza, and 12 in pacal. Each sampling station was in place for two days and was baited one day with 15 *P. macrostachya* seeds, the other day with tunafish and strawberry jam. Protein and sugar baits are common and reproducible means of assessing overall ant diversity at a site [48] and we expected tuna and jam to attract potential seed predators as well as potential dispersers, which are often generalist or even predatory ants [47], [49]. The order of bait presentation was randomized. Baits were presented on 4.3 cm^2^ perforated plastic trays held in place with wire anchors. Baits were first set out in the morning about 0700 hours and replenished throughout the experiment. Ants were observed and collected at the baits three times throughout the day over the course of 12–14 hours: once in the morning (by 0900), once in the afternoon (between 1300 and 1600), and once after dark (between 1930 and 2100, using red-filtered light). At each seed bait, number of seeds removed since the previous visit was noted. Where seeds had been removed, or where ants were present at seed baits, the sampling station was observed for 10–20 min and re-visited again about 30 min later. Ants were sorted and identified to species or morphospecies, and specimens are deposited at the Universidad Nacional de San Antonio Abad del Cusco in Cusco, Perú. We tabulated a 2×2 contingency table in which ants were categorized as either *C. femoratus* or not *C. femoratus*. For each of these two classes, we counted the number of bait stations at which ants visited seeds, and the number of stations at which they visited food but not seeds. We used the FREQ procedure in SAS version 9.1.3 to perform a chi-square test that compared the conditional probability of *C. femoratus* appearing at seeds given its presence at a bait station (food or seeds) to the same conditional probability for the class of all other ants combined.

### Exclusion experiment

To further examine factors affecting dispersal and predation of *P. macrostachya* seeds, we conducted an ant and vertebrate exclusion experiment. Trials were conducted in a randomized complete block split plot design with four blocks, two plots per block, and four treatments per plot. The two plots in a block were in the same habitat type and were monitored on the same days, but one was within *C. femoratus* foraging territory (as previously determined by *C. femoratus* presence at food and seed baits) and one was not. Plots were 1 m^2^, and each included four treatments positioned at the four corners of the plot: exclusion of both vertebrates and ants with wire mesh cages and Tanglefoot (Tanglefoot Co., Grand Rapids, MI); exclusion of vertebrates with cages only; exclusion of arthropods with Tanglefoot only; and no exclusion (Fig. 3). For each treatment, 15 seeds were placed in a 4.3 cm^2^ perforated plastic tray that was glued in the center of a 13 cm diameter perforated plastic plate and secured to the forest floor with wire anchors. To exclude walking arthropods, Tanglefoot was spread in a 3–4 cm band around the plate perimeter. For vertebrate exclusion, cages (15 cm square, 7.5 cm high, made of 1.5 cm wire mesh) were secured over the plates with wire anchors. Initial placement of the four treatments within a plot was randomized. Twenty four hours later, seeds were counted and replaced, and the positions of treatments were rotated so that over the course of 4 days, a total of 60 seeds were subjected to each treatment, and each treatment experienced each position within the plot.

The number of seeds removed from each treatment in each plot was summed over the four days. To test for effects of *C. femoratus* and exclusion of vertebrates or arthropods on seed removal, seed counts were first subjected to the empirical logistic transformation to achieve homogeneity of variance [50]. The MIXED procedure in SAS 9.1.3 was used to fit a mixed model with fixed effects for the whole plot factor AG ants and the split-plot factors Tanglefoot and cage, and random effects of block and block×AG ant interaction. Because there was a significant interaction between Tanglefoot and AG ants, the simple effect of AG ants was tested separately in the presence and absence of Tanglefoot. The Satterthwaite option was used within the MIXED procedure because *F*-ratios for these simple effects were constructed using error terms that were linear combinations of multiple mean squares from the ANOVA table [51].

### Seed fate in AG carton

To estimate the survival success of seeds retrieved to AG carton, we censused *P. macrostachya* plants in 10 AGs occupied by *C. femoratus*, scoring individuals as seedlings (cotyledons only), established juvenile plants (mature leaves but no reproductive structures), or adult plants (reproductive structures present). Censuses were conducted in December 2006, near the end of *P. macrostachya* fruiting season, which lasts 2–3 months in the late dry season and early rainy season [52, E. Y. personal observation]. We censused gardens of which we had an unobstructed view, or which had recently fallen to an accessible height. Each garden was censused once. To estimate seed survival based on these data, we made three assumptions, supported by the following observations. First, *P. macrostachya* seeds have a very high germination rate once they contact a moist substrate, even if that substrate is inappropriate. We have observed them to germinate within a few days on the ground beneath AGs, on seed trays left out after the conclusion of experiments, and in AG carton samples kept in a plastic box with or without ants. We therefore assumed that all seeds retrieved to an AG would sprout to the seedling stage. Second, we have not found seed caches within gardens despite opening many nests. We therefore assume that all seedlings on an AG represent seeds collected during the same fruiting season. Finally, we assume that visible seedlings represented the sum of the season's seed-collecting, i.e., that seedlings had 100% survival during the months of *P. macrostachya* fruiting that led up to the census. While highly speculative at this point, these assumptions provide the foundation for the only available estimate of seed success in the AG system, and all assumptions are designed to give an upper bound to the possible range of seed survival rates in *P. macrostachya*. Given these assumptions, we used the observed snapshot ratio of adult plants to seedlings to estimate the maximum probability that a seed retrieved to an AG matures to an adult plant.
